# Domperidone Inhibits *Clostridium botulinum* C2 Toxin and *Bordetella pertussis* Toxin

**DOI:** 10.3390/toxins15070412

**Published:** 2023-06-25

**Authors:** Jinfang Jia, Maria Braune-Yan, Stefanie Lietz, Mary Wahba, Arto T. Pulliainen, Holger Barth, Katharina Ernst

**Affiliations:** 1 Institute of Experimental and Clinical Pharmacology, Toxicology and Pharmacology of Natural Products, Ulm University Medical Center, 89081 Ulm, Germany; 2 Institute of Biomedicine, University of Turku, FI-20520 Turku, Finland

**Keywords:** AB-type protein toxin, ADP-ribosylating toxin, pertussis toxin, whooping cough, C2 toxin, membrane transport, chaperone, approved drug

## Abstract

*Bordetella pertussis* toxin (PT) and *Clostridium botulinum* C2 toxin are ADP-ribosylating toxins causing severe diseases in humans and animals. They share a common translocation mechanism requiring the cellular chaperones Hsp90 and Hsp70, cyclophilins, and FK506-binding proteins to transport the toxins’ enzyme subunits into the cytosol. Inhibitors of chaperone activities have been shown to reduce the amount of transported enzyme subunits into the cytosol of cells, thus protecting cells from intoxication by these toxins. Recently, domperidone, an approved dopamine receptor antagonist drug, was found to inhibit Hsp70 activity. Since Hsp70 is required for cellular toxin uptake, we hypothesized that domperidone also protects cells from intoxication with PT and C2. The inhibition of intoxication by domperidone was demonstrated by analyzing the ADP-ribosylation status of the toxins’ specific substrates. Domperidone had no inhibitory effect on the receptor-binding or enzyme activity of the toxins, but it inhibited the pH-driven membrane translocation of the enzyme subunit of the C2 toxin and reduced the amount of PTS1 in cells. Taken together, our results indicate that domperidone is a potent inhibitor of PT and C2 toxins in cells and therefore might have therapeutic potential by repurposing domperidone to treat diseases caused by bacterial toxins that require Hsp70 for their cellular uptake.

## 1. Introduction

AB-type protein toxins are the most potent toxins known today. They are the cause of serious human and animal diseases and can be used as biological weapons. AB-type toxins comprise, for example, the *Bacillus anthracis* anthrax toxin, *Bordetella (B.) pertussis* toxin (PT), *Corynebacterium diphtheriae* toxin (DT), *Vibrio cholerae* toxin (CT), and different clostridial binary toxins, including *Clostridium (C.) botulinum* C2 toxin. All these toxins consist of a binding/transporting B subunit and one (or two in the case of anthrax toxins) enzymatically active A subunits.

As the prototype of clostridial binary toxins, C2 toxin is composed of an enzyme A component C2I and a separate binding/translocation B component C2II [1,2]. The proteolytic activation of C2II results in biologically active C2IIa that forms ring-shaped heptamers, which bind to asparagine-linked carbohydrate structures located on the surface of all cell types that have been investigated so far [3,4]. C2I attaches to the C2IIa heptamers, and the C2IIa/C2I complex is taken up into cells via receptor-mediated endocytosis [5,6,7]. The acidification of the endosomal lumen leads to conformational changes in C2IIa heptamers, which results in the formation and insertion of a C2IIa pore into the endosomal membrane [3,8]. C2I partially unfolds to translocate through the narrow C2IIa pore into the target cell’s cytosol [8,9]. In the cytosol, C2I refolds and mediates the covalent transfer of an ADP-ribose moiety from the co-substrate NAD^+^ onto G-actin [10]. The C2I-mediated mono-ADP ribosylation of G-actin turns G-actin into a capping protein that prevents the further addition of G-actin to the growing end of actin filaments [11]. This results in the depolymerization of F-actin and the rounding of adherent cells, which leads to a breakdown of the gut barrier and causes characteristic clinical symptoms, i.e., enterotoxicity [1,2,12].

PT is an AB_5_ toxin consisting of an enzyme subunit, the A protomer PTS1, and a pentameric binding/transport B subunit which is formed by PTS2, PTS3, PTS4, and PTS5 in a ratio of 1:1:2:1 [13,14]. PTS1 associates with the B pentamer through non-covalent bonds in the periplasm of *B. pertussis* as a PT holotoxin with a pyramid-like structure and is then secreted by a type-IV secretion system [14,15]. The B pentamer facilitates the binding of PT to sialoglycoproteins on the surface of target cells [16]. PT, as an intact holotoxin, is internalized by receptor-mediated endocytosis and follows a retrograde intracellular transport through the Golgi apparatus to the endoplasmic reticulum (ER) [17,18,19]. In the ER, ATP binds the central pore of the B pentamer, leading to a structural change in the B pentamer, which contributes to the release of PTS1 [20,21]. Released PTS1 is thermally unstable and consequently shifts to a disordered state [22]. Unfolded PTS1 is subsequently recognized as an ER-associated degradation (ERAD) substrate for export from the ER into the cytosol [22,23]. PTS1 lacks lysine residues, which are required for the ubiquitination of proteins and therefore it is protected from subsequent ubiquitin-dependent degradation by the proteasome [24]. In the cytosol, PTS1 refolds and covalently transfers an ADP-ribose moiety from the co-substrate NAD^+^ onto the α-subunit of inhibitory G proteins (Gαi), which results in the inability of Gαi to bind to G-protein-coupled receptors (GPCRs) [25,26]. Accordingly, the agonist stimulation of GPCRs becomes ineffective, which, for example, is manifested as an increased level of cAMP due to the lack of the Gαi-mediated inhibition of adenylate cyclase (AC) activity [27]. PT is an important virulence factor of pathogenic *B. pertussis* and plays an etiological role in causing pertussis (whooping cough) [28,29,30]. Despite widespread vaccine coverage, its incidence is at its highest level since the introduction of the pertussis vaccination in the 1950s [31]. Antibiotics can be used to prevent the spread of the disease, but are only effective if administered in very early stages of infection and in most cases do not improve severe symptoms [32,33]. The re-emergence of whooping cough and the lack of effective treatments, as well as the importance of PT in disease severity, suggest that targeting PT is an effective strategy for drug development.

Although the uptake mechanisms of C2 toxin and PT are not identical, a common trans-membrane translocation mechanism involving the requirement of host cell chaperones (Hsp90 and Hsp70) and peptidyl-prolyl cis/trans isomerases (PPIases) of the cyclophilin (Cyps) and FK506-binding protein (FKBPs) families is evident [34,35,36,37,38,39,40,41,42,43,44]. The identification of commonalities in the mechanism of action of these toxins could aid in the development of therapeutics that work against different bacterial species. Thus, trans-membrane translocation has provided an attractive starting point for new pharmacological strategies over the past few years [45,46,47]. For investigating the role of host cell chaperones, specific pharmacological inhibitors of chaperone activities are used. The inhibitor VER-155008 (VER), an inhibitor of Hsp70 and Hsc70, binds with a high affinity to the ATP-binding site of Hsp70 or Hsc70, thereby inhibiting their activities [48]. Despite the effectiveness of VER in protecting cells from Hsp/Hsc70-assisted intoxication by C2 toxin, PT, and several other AB-type toxins, its transition into clinical use may be lengthy due to the substantial time and cost of clinical studies. Recently, the licensed drug domperidone (DOM) was discovered as a novel Hsp70-inhibitor in a study aiming to find FDA-approved drugs that are structurally similar to another Hsp70-inhibitor, HS-72 [49]. Here, a set of experiments was performed to prove if domperidone also protects cells from intoxication with C2 toxin and PT.

## 2. Results

### 2.1. Domperidone Protects Cells from Intoxication with C2 Toxin

**Figure 1:**
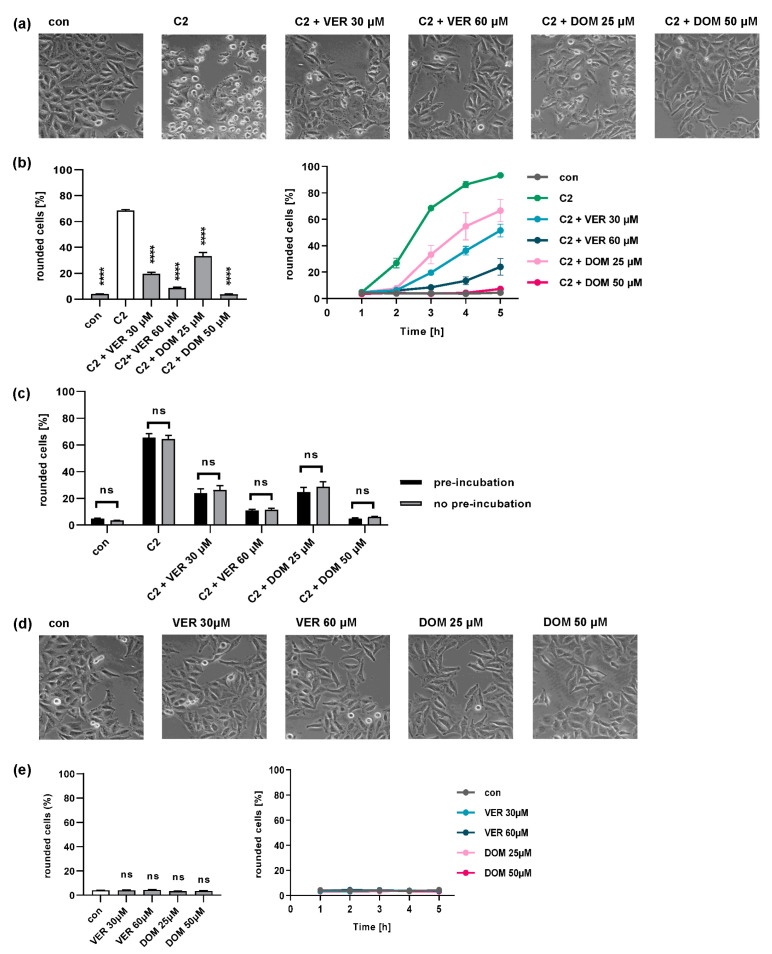
DOM protects HeLa cells from intoxication by C2 toxin. (**a**) HeLa cells were pre-incubated at 37 °C with VER or DOM for 30 min or left untreated as the control (con). Then, cells were challenged with C2 toxin (50 ng/mL C2I plus 100 ng/mL C2IIa) for 5 h in the presence or absence of the respective inhibitors. Pictures show the toxin-induced morphological changes after 3 h. (**b**) The percentage of rounded cells was determined from images after 3 h (left) and at indicated time points (right) (n = 4, mean ± SEM, n ≥ 24 values from 4 independent experiments). (**c**) HeLa cells were pre-incubated with DOM or VER or left untreated as the control for 30 min at 37 °C. Then, cells were challenged with C2 toxin (50 ng/mL C2I plus 100 ng/mL C2IIa) for 4 h in the presence or absence of the respective inhibitors. Rounded cells were counted as described above (n = 4, mean ± SEM). (**d**) HeLa cells were incubated with VER or DOM or left untreated as the control (con). Pictures show cell morphology after 3 h. (**e**) The percentage of rounded cells was determined from images after 3 h (left) and at indicated time points (right) (n = 4, mean ± SEM, n ≥ 24 values from 4 independent experiments). Significance was tested by mixed-effects analysis with multiple comparison test. Significance was tested against C2-toxin-treated samples (**b**) or untreated control samples (**e**) (white bars), (**** *p* < 0.0001, ns = not significant *p* > 0.05).

We showed previously that the pharmacological inhibition of Hsp70 activity by VER protects cells from intoxication with C2 toxin [38,39,40]. Here, we compare the inhibitory effect of VER and DOM on the C2 toxin intoxication of cells. Therefore, an intoxication assay was performed, which employed a morphology-based readout of adherent cells, i.e., the rounding of HeLa cells due to the destruction of the actin cytoskeleton by C2 toxin. VER and DOM both markedly delayed the intoxication of HeLa cells with C2 toxin in a time- and concentration-dependent manner (Figure 1a,b). We established the concentrations used in this study based on previous experiments, which consistently demonstrated the most reliable inhibitory effect (Supplementary Figure S1) [38,42]. The inhibitory effects of VER and DOM on C2 toxin intoxication did not vary with or without the pre-incubation of cells with the inhibitors (Figure 1c). This suggests that the simultaneous addition of an inhibitor and toxin provides the inhibitor with sufficient time to inhibit Hsp70 within the cells, thereby preventing intoxication, which could be beneficial in a potential therapeutic setting. Cells treated with VER or DOM alone did not change their morphology compared to untreated control cells (Figure 1d,e).

**Figure 2:**
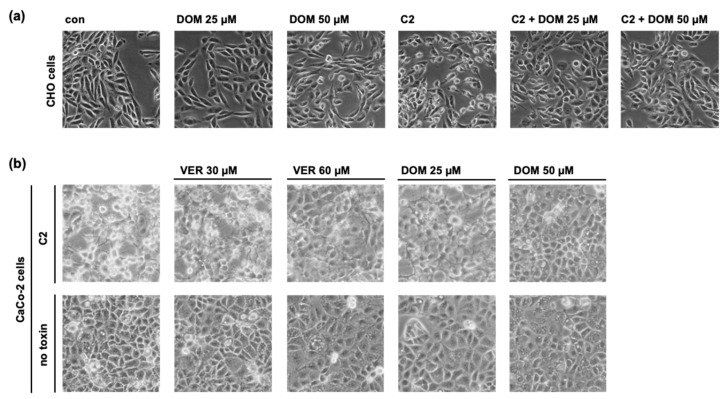
DOM protects CHO cells as well as human colon carcinoma CaCo-2 cells from C2 intoxication. CHO cells (**a**) or CaCo-2 cells (**b**) were pre-incubated with DOM or VER for 30 min or left untreated as the control. C2 toxin ((**a**) 50 ng/mL C2I plus 100 ng/mL C2IIa; and (**b**) 100 ng/mL C2I plus 200 ng/mL C2IIa) was added, and cell morphology was monitored every hour. The control cells were treated with inhibitors only. Images show cell morphology 3 h (**a**) and 5.5 h (**b**) after intoxication.

To investigate the intoxication with the C2 toxin, HeLa cells are used as the primary experimental model because the morphological effects induced by the toxin in these cells provide a robust readout. Thus, in this study, we also used HeLa cells in most of the experiments to uncover the mechanisms of inhibition by DOM. However, the protective effect of DOM on intoxication with C2 toxin was also observed for CHO cells as well as for the human colon carcinoma cell line CaCo-2, which represents a pathophysiologically more relevant cell line in the context of the enterotoxicity observed by the C2 toxin (Figure 2).

### 2.2. Domperidone Reduces the Amount of ADP-Ribosylated G-Actin in Cells without Affecting the C2I Enzyme Activity In Vitro or the Binding of C2 Toxin to Cells

**Figure 3:**
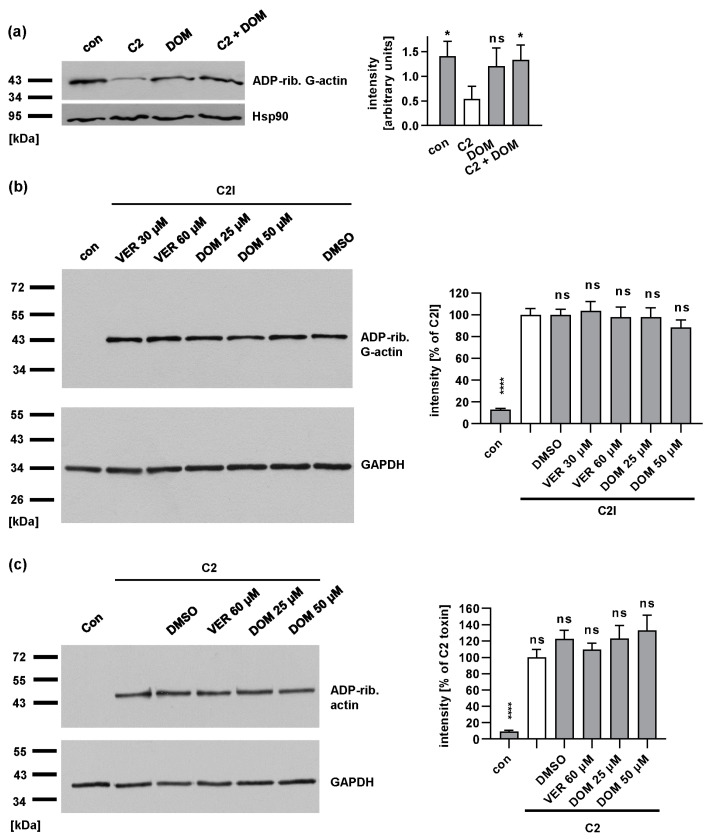
DOM reduces the amount of ADP-ribosylated G-actin in cells without affecting C2I enzyme activity in vitro or binding of C2 toxin to cells. (**a**) HeLa cells were pre-incubated with 50 µM DOM for 30 min. C2 toxin (100 ng/mL C2I plus 200 ng/mL C2IIa) was added for 5 h. Cells were lysed and incubated with fresh C2I in the presence of biotin-labeled NAD^+^. ADP-ribosylated, biotin-labeled G-actin was detected by Western blotting. Comparable protein loading was confirmed by Hsp90 detection. Intensity of signals was quantified. Values were normalized to Hsp90 loading control (n = 3 values from 3 independent experiments, mean ± SD). Significance was tested by one-way ANOVA with Dunnetts’s multiple comparison test. Significance was tested against C2 toxin-treated samples (white bar). (**b**) HeLa cell lysate was pre-incubated with VER or DOM or left untreated as the control for 30 min at 37 °C. For further control, cell lysate was incubated with DMSO, the solvent of VER and DOM. The final concentration of DMSO corresponded to the highest DMSO concentration used for inhibitors. Subsequently, 100 nM C2I and biotin-NAD^+^ were added for 30 min at 37 °C. Biotin-labeled (i.e., ADP-ribosylated) G-actin was detected by Western Blot analysis using streptavidin–peroxidase. Comparable protein loading was confirmed by GAPDH detection. Intensity of signals for ADP-ribosylated G-actin were quantified. Values show the percentage of signal intensity referring to samples treated with toxin only and are normalized on the respective loading control obtained by GAPDH detection. Values are given as mean ± SEM (n ≥ 12 values from 4 independent experiments). Significance was tested against samples treated with C2I only by mixed-effects analysis and multiple comparisons test (white bar). (**c**) HeLa cells were pre-incubated with VER or DOM for 30 min. C2 toxin (100 ng/mL C2I plus 200 ng/mL C2IIa) was added at 4 °C for 40 min. The cells were treated with DMSO, solvent of inhibitors, or left untreated (con). After washing, cells were lysed, and bound C2 toxin was detected by measuring the C2I enzyme activity in the presence of biotin-NAD^+^. GAPDH was detected as loading control. Signal intensities were quantified and normalized to GAPDH and then to samples treated with C2 toxin only (white bar). Values are given as mean ± SEM (n ≥ 7 values from 4 independent experiments). Significance was tested against samples treated with C2 toxin only by mixed-effects analysis and multiple comparisons test, (**** *p* < 0.0001, * *p* < 0.05, ns = not significant *p* > 0.05).

To confirm the inhibitory effect of DOM, the intracellular substrate modification by C2I was assessed by a Western blot analysis. Lysates from C2 toxin-treated cells with or without DOM were incubated with C2I in the presence of biotin-labeled NAD^+^. Thereby, the G-actin that had not been ADP-ribosylated during the intoxication of intact cells was ADP-ribosylated in vitro and thus biotin labeled. ADP-ribosylated and therefore biotin-labeled G-actin was detected by Western blotting. Figure 3a shows a weak signal for the C2-toxin-treated samples compared to that of the untreated controls, indicating ADP-ribosylation in the intact cells. In the presence of DOM, the signal for C2-toxin-treated cells is stronger, demonstrating inhibited intoxication.

The morphological as well as substrate modification analyses clearly indicated the inhibitory effect of DOM on intoxication by the C2 toxin. To explain this, we developed the following hypotheses: (1) DOM reduces the enzyme activity of C2I; (2) DOM reduces the binding of C2 toxin to cells; or (3) DOM interferes with the transport of C2I into the cytosol. To address the first hypothesis, HeLa cell lysates were incubated with or without VER or DOM for 30 min before the addition of C2I in the presence of biotin-labeled NAD^+^. Biotin-labeled (i.e., ADP-ribosylated) G-actin was then detected by Western blotting. As shown in Figure 3b, a comparable amount of biotin-labeled actin was detected if the samples were treated with inhibitors, compared to that of the samples treated with C2I alone, demonstrating that VER and DOM do not inhibit the enzyme activity of C2I.

To analyze if DOM inhibits the binding of C2 toxin on the cell surface, cells were incubated with the C2 toxin at 4 °C with or without DOM to allow for binding to the cells but not the uptake of the C2 toxin by endocytosis. Bound C2 toxin was detected by assessing the enzyme activity of C2I from the cell lysates using biotin-labeled NAD^+^. DOM had no significant effect on the binding of the C2 toxin to the cells (Figure 3c).

### 2.3. Domperidone Inhibits the pH-Driven Membrane Translocation of C2I into the Cytosol

**Figure 4:**
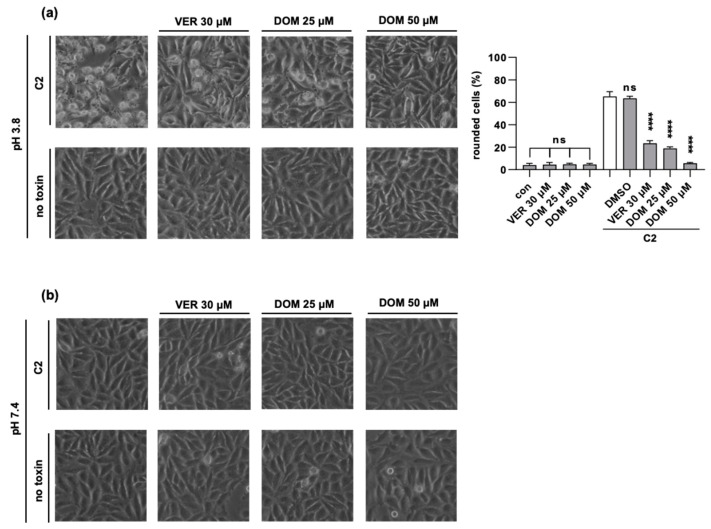
DOM inhibits pH-dependent translocation of C2I into the cytosol. HeLa cells were pre-incubated with inhibitors for 30 min. BafA1 was added for 30 min to all samples, and then cells were incubated on ice with C2 toxin for 30 min. For the control, cells were treated with inhibitors only or left untreated (images in the lower row). Warm acidic medium was added for 10 min and then replaced with fresh medium (**a**). For the control, warm neutral medium was added for 10 min and then replaced with fresh medium (**b**). Cells were further incubated at 37 °C, and cell morphology was monitored. Images show cells after 4 h of incubation with C2 toxin. Percentage of rounded cells was quantified from images. Values are given as mean ± SD (n = 3 values from 1 representative of 3 independent experiments). Significance was tested by one-way ANOVA and Dunnett’s multiple comparisons test and refers to samples treated with C2 toxin only (white bar) if not indicated otherwise, (**** *p* < 0.0001, ns = not significant *p* > 0.05).

After excluding an inhibitory effect on the cell binding or enzyme activity of DOM, we tested whether the membrane translocation of C2I is affected by DOM. Therefore, the acidic endosomal conditions required for the translocation of C2I were mimicked directly at the cytoplasmic membrane [3]. This assay was first established by Sandvig et al. in 1980 to investigate the pH-dependent translocation of diphtheria toxin [50]. It was then adapted for other short-trip toxins, such as anthrax toxin, *Clostridioides difficile* TcdB toxin, as well as C2 toxin [3,51,52]. In this assay, C2 toxin was allowed to bind to the cell surface on ice, for which endocytosis was blocked due to the cold temperature. Additionally, bafilomycin A1 (BafA1) was used to block the acidification of endosomes and thus the translocation of C2I from endosomes into the cytosol. By the addition of a warm acidic medium to the cells, pore formation by C2IIa heptamers and the translocation of C2I across the cytoplasmic membrane were triggered (Figure 4a). If a warm neutral medium (pH 7.4) was added, C2I did not cross the membrane, and the cells did not round up (Figure 4b). Thereby, the effect of DOM on membrane translocation was investigated in an isolated manner, and we showed that DOM inhibited this crucial step of C2 toxin uptake into the cytosol (Figure 4).

### 2.4. Domperidone Reduces the Amount of ADP-Ribosylated Gαi in PT-Treated Cells without Interfering with the Enzyme Activity In Vitro or the Cell Binding of PT to Cells

**Figure 5:**
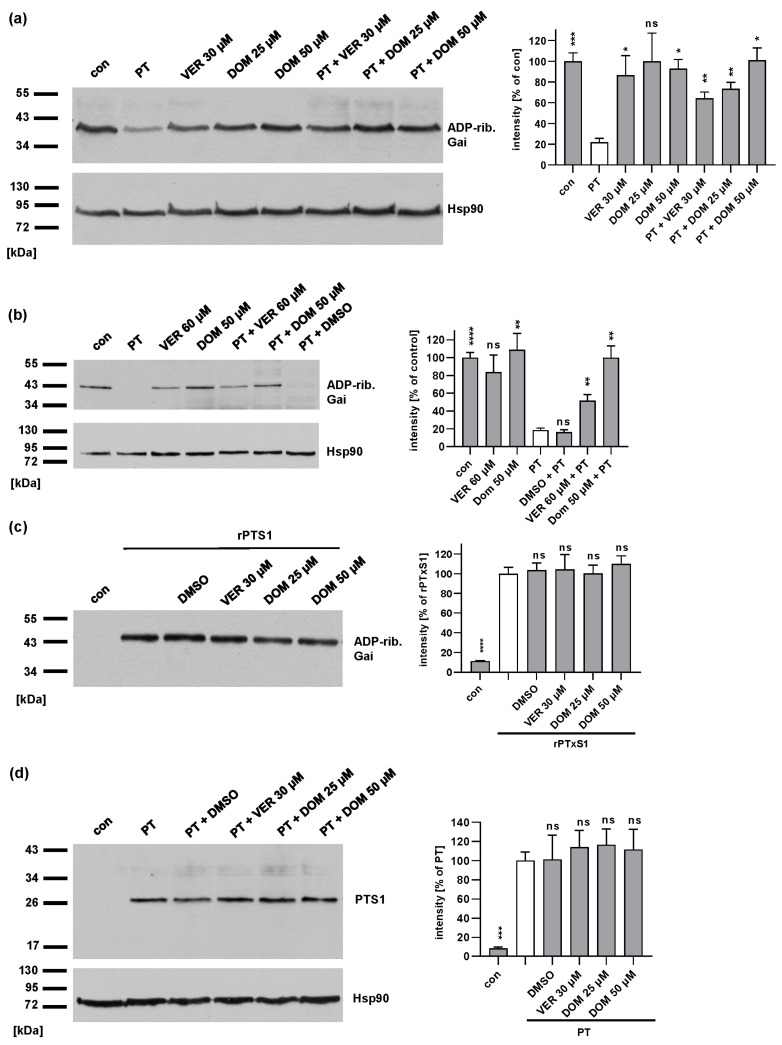
DOM reduces the amount of ADP-ribosylated Gαi in cells without affecting PTS1 enzyme activity in vitro or binding of PT to cells. (**a**) CHO cells were pre-incubated with VER or DOM for 30 min at 37 °C. As the control, cells were left untreated (con). Then, cells were challenged with 10 ng/mL PT for 4 h in the presence or absence of the respective inhibitors. Cells were lysed and the ADP-ribosylation status of Gαi from these cells was analyzed by incubation with 100 nM PTS1 in the presence of biotin-NAD^+^. Biotin-labeled (i.e., ADP-ribosylated) Gαi was detected. Comparable protein loading was confirmed by Hsp90 detection. Values show the percentage of signal referring to untreated cells and are normalized on the respective loading control obtained by Hsp90 detection, given as mean ± SEM (n ≥ 4 values from 4 independent experiments). Significance was tested by mixed-effects analysis, and values refer to samples treated with PT only (white bar). (**b**) A549 was pre-incubated with VER or DOM or used as the control with DMSO for 30 min at 37 °C. A total of 10 ng/mL PT was added to cells for 4 h in the presence or absence of the respective inhibitors. Subsequently, cells were lysed, and the ADP-ribosylation status of Gαi from these cells was analyzed. Biotin-labeled (i.e., ADP-ribosylated) Gαi was detected. Comparable protein loading was confirmed by Hsp90 detection. Values show the percentage of signal referring to untreated cells are normalized on the respective loading control obtained by Hsp90 detection and are given as mean ± SEM (n ≥ 6 values from 5 independent experiments). Significance was tested by using the mixed-effects analysis and Dunnett’s multiple comparisons test, and values refer to samples treated with PT only (white bar). (**c**) Recombinant Gαi was pre-incubated with VER or DOM or used as the control with DMSO (solvent of inhibitors) for 30 min at room temperature. For further control, recombinant Gαi was incubated with buffer only. Subsequently, 100 nM rPTS1 and 10 µM biotin-labeled NAD^+^ were added and incubated for 30 min at room temperature. Biotin-labeled (i.e., ADP-ribosylated) Gαi was detected with streptavidin–peroxidase, and intensity of signals was quantified by densitometry. Values are given as percent of samples treated with rPTS1 only (mean ± SEM, n ≥ 6 values from 3 independent experiments). Significance was tested by mixed-effects analysis and Dunnett’s multiple comparisons test and values refer to samples treated with rPTS1 only (white bar). (**d**) CHO cells were pre-incubated with VER or DOM or used as the control with DMSO for 30 min at 37 °C. A total of 500 ng/mL PT was added to cells at 4 °C for 40 min. After washing, bound PT was detected by Western blotting using a specific antibody against PTS1. Equal loading was confirmed by Hsp90 detection. Western blot signals were quantified and normalized to Hsp90 signals and then to samples treated with PT only (mean ± SEM, n ≥ 4 values from 4 independent experiments). Significance was tested by mixed-effects analysis and Dunnett’s multiple comparisons test and values refer to samples treated with rPTS1 only (white bar), (**** *p* < 0.0001, *** *p* < 0.001, ** *p* < 0.01, * *p* < 0.05, ns = not significant *p* > 0.05).

Since the cellular uptake of PT also depends on the activity of Hsp70 [42], we tested if DOM also inhibits the intoxication of cells with PT. CHO cells (Figure 5a) were investigated because intoxication with PT can be detected in reproducible and reliable manner in these cells. Moreover, CHO cells are also used to detect residual PT activity in the vaccine manufacturing process [53]. Human lung epithelial A549 cells (Figure 5b) were used because they represent a pathophysiologically more relevant cell line since whooping cough mainly affects the respiratory tract. The cells were treated with PT in the presence or absence of VER or DOM and were subsequently lysed. Then, the cell lysates were incubated with fresh PTS1 in the presence of biotin-labeled NAD^+^, allowing the biotin-labeling of Gαi that had not been ADP-ribosylated during the intoxication of intact cells. Figure 5a,b reveal that strong signals were obtained from the control samples, whereas weaker signals were observed in the presence of PT. This indicates that a substantial amount of Gαi was already ADP-ribosylated in the PT-treated cells and could not serve as substrate for the subsequent in vitro ADP-ribosylation. The pre-treatment of the cells with VER or DOM prior to the PT intoxication reduced the ADP-ribosylation of Gαi. This result demonstrates that VER and DOM protected the living cells from intoxication with PT. The treatment of cells with inhibitors alone did not affect the subsequent in vitro ADP-ribosylation of Gαi by PTS1 (Figure 5a,b). Moreover, we were able to prove that the reduced ADP-ribosylation of Gαi by PTS1 in the presence of inhibitors did not result from the inhibition of the enzyme activity of PTS1. Figure 5c demonstrates that a comparable amount of biotin-labeled recombinant Gαi was detected by Western blotting if the samples were treated with inhibitors compared to the samples treated with the recombinant PTS1 alone. To analyze if DOM inhibits the binding of PT on the cell surface, cells were incubated with PT at 4 °C with or without DOM to allow binding to cells but not the uptake of PT by endocytosis. The Bound PT was detected from the cell lysates by the Western blot of PTS1. DOM had no inhibitory effect on the binding of PT to the cells (Figure 5d).

### 2.5. In the Presence of Domperidone, Less Free PTS1 Is Detectable in Cells

**Figure 6:**
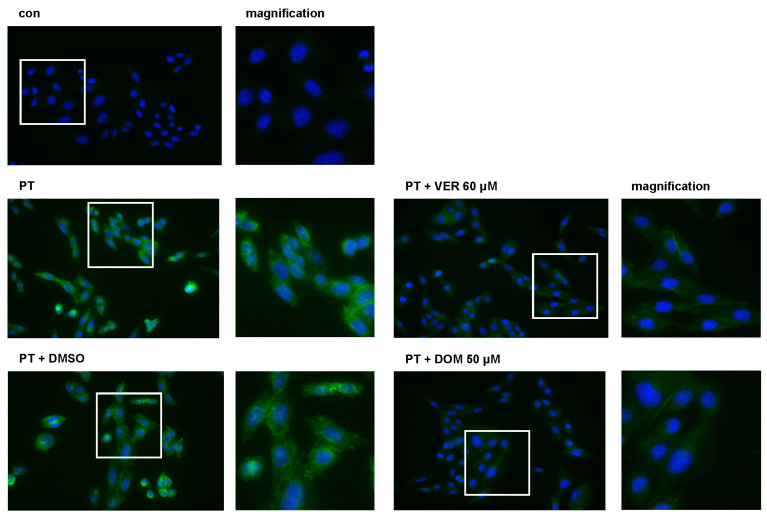
In the presence of DOM, less free PTS1 is detected in cells. CHO cells were pre-incubated with the respective inhibitors for 30 min at 37 °C. As the control, cells were treated with DMSO, the solvent of inhibitors, or left untreated. PT (100 ng/mL) was added for 4 h. After washing, cells were fixed and permeabilized, and PTS1 was stained by incubation with a specific primary antibody followed by fluorescence-labeled secondary antibody. Nuclei were stained by Hoechst. Images were taken with Keyance fluorescence microscope. Green = PTS1; blue = nucleus. White squares indicate magnified area.

We used a specific antibody against PTS1 that preferably recognizes PTS1 when it is detached from the B pentamer in cells representing mostly cytosolic PTS1 [41,42]. The fluorescence signals of PTS1 were reduced in the samples treated with DOM or VER compared to that of the samples treated only with PT or with DMSO and PT (Figure 6). These results suggest that DOM is comparable to VER and interferes with the uptake of PTS1 into the cytosol of cells.

### 2.6. Inhibitors Reduce the Chaperone-Mediated Increase in PTS1 Enzyme Activity

**Figure 7:**
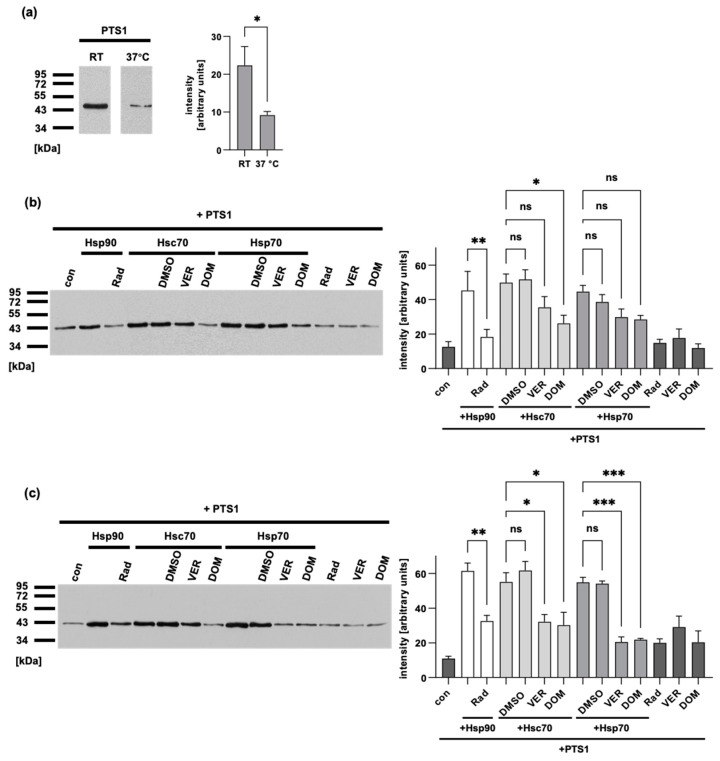
Inhibitors reduce the chaperone-mediated increase in PTS1 enzyme activity. (**a**) Recombinant PTS1 (100 nM) was incubated at RT or 37 °C for 15 min. ATP (1 mM) was added for another 30 min followed by addition of recombinant Gαi (0.8416 µM) and 10 µM biotin-NAD^+^ for 30 min. Biotin-labeled (i.e., ADP-ribosylated) Gαi was detected by Western blotting. Western blot signals were quantified and are shown as a bar graph. Values are given as mean ± SD (n = 3 values of 3 independent experiments). Significance was tested by Welch’s *t*-test. Western blot images were cropped for display purposes only. (**b**,**c**) Recombinant PTS1 (100 nM) was incubated at RT (**b**) or 37 °C (**c**) for 15 min. Meanwhile, ATP (50 µM) and chaperones (200 nM Hsp90/Hsc70, 50 nM Hsp70) were pre-incubated for 15 min at RT or 37 °C before being added to PTS1 and further incubated. After 30 min, recombinant Gαi (0.8416 µM) was added to 10 µM biotin-NAD^+^ and incubated for 30 min at RT or 37 °C. Biotin-labeled (i.e., ADP-ribosylated) Gαi was detected by Western blotting. Western blot signals were quantified and are shown as a bar graph. Values are given as mean ± SEM (n = 6 values of 4 independent experiments (**b**) or n = 4 values of 2 independent experiments (**c**)). Significance was tested by one-way ANOVA and Tukey’s multiple comparisons test. Western blot images show results of one representative experiment, (*** *p* < 0.001, ** *p* < 0.01, * *p* < 0.05, ns = not significant *p* > 0.05).

PTS1 and CtxA, the ADP-ribosyltransferase subunit of cholera toxin, are both thermally unstable, which means that the enzymes unfold at temperatures of 37 °C [22,54]. Previous studies have also demonstrated that Hsp90 helps CtxA to regain enzyme activity faster after thermal unfolding [22,23,55]. Here, we show that the enzyme activity of PTS1 is reduced at 37 °C compared to that at RT (Figure 7a). Subsequently, we conducted experiments in which PTS1 was incubated at RT or 37 °C followed by incubation with Hsp90, Hsp70, or Hsc70. Then, Gαi and NAD^+^ were added, and the enzyme activity was measured by the detection of ADP-ribosylated Gαi. The results showed an increase in enzyme activity in the presence of the respective chaperones at RT and 37 °C (Figure 7b,c). Furthermore, the specific inhibitors of Hsp90 and Hsp/c70, radicicol (Rad) and VER/DOM, respectively, prevented this increase (Figure 7b,c). This indicates that the chaperones assisted the enzyme activity of PTS1 and that the ATPase activity of the chaperones is required.

### 2.7. Domperidone Reduces the PT-Mediated Effects on cAMP Signaling

**Figure 8:**
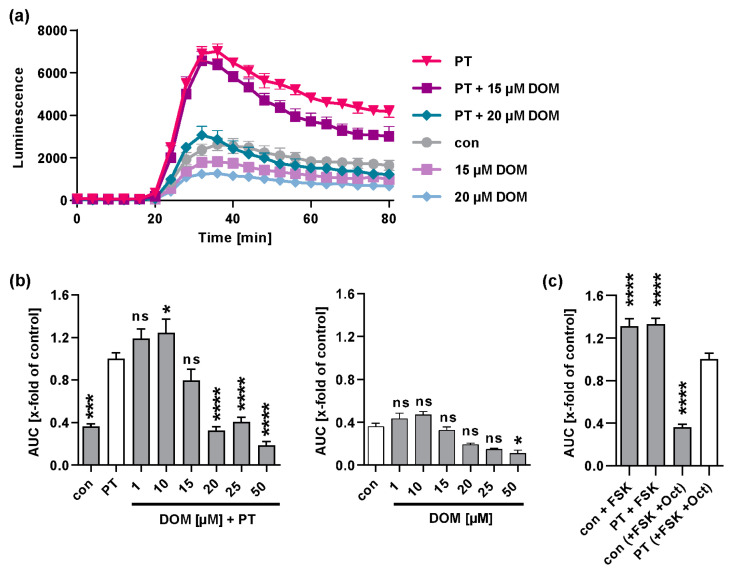
DOM reduces the PT-mediated effects on cAMP signaling. Cells were treated with DOM at indicated concentrations and PT (100 ng/mL) for 5 h at 37 °C or cells were left untreated (con). Inducing medium with luciferase substrate for the luminescent cAMP biosensor was added. Baseline was measured for 15 min, then cells were spiked with forskolin (FSK, activator of adenylate cyclase) and octreotide acetate (Oct, activator of SSTR2). Luminescence was recorded for 60 min. (**a**) cAMP kinetic curves of one representative experiment are shown. Values are given as mean ± SD, n ≥ 3 from one experiment. (**b**) Bar graph shows baseline-subtracted area under the curve (AUC) from at least three independent experiments. Values are given as percent of samples treated with PT only, mean ± SEM, n ≥ 6. (**c**) As the control, cells were treated with FSK or PT plus FSK without Oct to detect maximal cAMP response. Values for the control samples and samples treated with PT, FSK, and Oct are identical to values in (**b**). Significance was tested by one-way ANOVA analysis and Dunnett’s multiple comparisons test, and values refer to samples treated with PT only (left graph in (**b**,**c**), white bar) or con (right graph in (**b**), white bar), (**** *p* < 0.0001, *** *p* < 0.001, * *p* < 0.05, ns = not significant *p* > 0.05).

In the cytosol, the PTS1-mediated ADP-ribosylation of Gαi results in the inability of Gαi to bind to GPCRs [25,26]. Accordingly, the agonist stimulation of inhibitory GPCRs becomes inefficient, i.e., Gαi is no longer able to efficiently inhibit the adenylate cyclase. To assess the effect of PT on cAMP signaling in living cells, an iGIST bioassay was used [27]. The assay utilizes HEK293 cells that ectopically express the Gαi-coupled GPCR somatostatin receptor 2 (SSTR2) and a luminescent cAMP probe. The cells are treated with forskolin to activate the adenylate cyclase and with octreotide to activate SSTR2. SSTR2 stimulation lowers the cAMP levels due to the Gαi-mediated inhibition of the adenylate cyclase. PT is able to reverse this effect. In the iGIST bioassay, an inhibition of PT activity was observed when the cells were exposed to DOM concentrations starting from 15 µM (Figure 8). Although higher concentrations of DOM exhibited a slight decrease in the cAMP signal independently, it was only with samples treated with 50 µM DOM that a significant reduction was observed. A minor increase in cAMP levels was observed when the cells were treated with 10 µM DOM and PT, compared to that of the cells treated only with PT (Figure 8b). However, this increase represents a relatively small effect in comparison to the more substantial inhibition observed with higher concentrations tested. Previous experiments have demonstrated an even stronger effect of VER when administered alone [42]. Consequently, VER was not included in this study. The maximum levels of cAMP were attained when the cells were treated solely with forskolin, and only a slight reduction was observed when the cells were treated with PT in the presence of forskolin plus octreotide (Figure 8c). The data imply that DOM is able to reduce the PT-mediated effect on host cell cAMP signaling. 

Taken together, we showed that DOM protects cells from intoxication with C2 toxin and PT, comparable to the established Hsp70 inhibitor VER. DOM reduced the number of rounded cells and the amount of ADP-ribosylated G-actin in C2 toxin-treated cells. In the PT-treated cells, DOM reduced the amount of ADP-ribosylated Gαi. However, DOM had no effect on the cell binding or enzyme activity in vitro of both toxins, but it impaired the membrane translocation of C2I into the cytosol and reduced the amount of cytosolic PTS1. DOM also reduced the chaperone-mediated increase in PTS1 enzyme activity in vitro and decreased the effect of PT on host cell cAMP signaling.

## 3. Discussion

Over the past few years, several groups including our own have investigated the membrane translocation of the enzyme subunits of bacterial AB-type toxins from intracellular compartments to the target cell cytosol [46]. *B. pertussis* mediates respiratory disease through its ADP-ribosylating toxin, PT. After internalization by endocytosis, PT reaches the ER through the Golgi via the retrograde intracellular pathway, and subsequently the disordered PTS1 is transported from the ER to the cytosol via the ER-associated degradation pathway [18]. Compared to PT, clostridial binary toxins, such as C2 toxin, take a more direct route into the cytosol. They are taken up by receptor-mediated endocytosis and then deliver their enzyme components from acidified endosomes to the cytosol, where they ADP-ribosylate their specific substrate molecules to cause severe enterotoxicity in humans and animals [56]. However, a prerequisite for the translocation of these toxins across membranes is an at least partial unfolding of their enzyme subunits [9,23]. Interestingly, the chaperones Hsp70, Hsp90, FKBP, and Cyps were identified as important interaction partners of the enzyme subunit of toxins, including the clostridial binary toxins C2 toxin, iota toxin, CDT toxin [34,35,36,37,38,39,40,57,58,59], diphtheria toxin [60,61,62], and pertussis toxin [41,42,43] during membrane translocation (for a review, see [45]).

Hsp70s are involved in a wide range of cellular processes, including protein folding, the assembly and disassembly of protein complexes, the regulation of signaling pathways, and protection against stressors such as heat, radiation, and oxidative stress [63]. One of the primary functions of Hsp70 is to assist in the folding of newly synthesized or denatured proteins. Hsp70 binds to hydrophobic regions of nascent or misfolded proteins, preventing their aggregation and promoting their proper folding. In addition, Hsp70 can also function as a co-chaperone, working in conjunction with other chaperones to facilitate the folding and assembly of specific client proteins. Hsp70 is also involved in the transport of proteins across cellular membranes by the mechanism of entropic pulling. Hsp70 binds tightly to the translocating protein close to the translocation pore. This results in a reduction in Brownian motion and generates a unidirectional pulling force, guiding the protein through the pore [64,65,66].

Notably, Hsp70 has been shown to act together with Hsp90, Cyps, and FKBPs in the folding and activation of steroid hormone receptors [67]. These same chaperones have been identified as assisting in the uptake of C2 toxin, PT, and other toxins, suggesting that they also act in a concerted manner to facilitate the membrane translocation of these toxins [45,46]. A screening of a compound library for compounds that structurally resemble the known Hsp70 inhibitor HS-72 resulted in the discovery that domperidone inhibits Hsp70 [49,68]. It was demonstrated that domperidone and HS-72 significantly reduced the ATPase activity of Hsp70, with domperidone showing stronger inhibition than HS-72 [49]. Domperidone is used to treat nausea and vomiting related to medical conditions such as gastroesophageal reflux disease [69]. Adverse effects, such as headache, dry mouth, dizziness, and gastrointestinal disturbances, have been reported. In rare cases, serious adverse effects, such as cardiac arrhythmias, have also been reported [69,70]. Whooping cough is treated primarily with antibiotics, which prevent transmission but rarely affect the course of disease because they are usually administered too late [31]. Although PT is strongly associated with the disease’s severity, for example, by most likely causing leukocytosis, the hallmark of severe pertussis, there are no PT-directed therapeutic strategies available [30,71]. Blocking Hsp70 activity is a potential strategy to counteract the uptake of PT into the cytosol and neutralize the toxin molecules already present inside the cells. As an approved drug, domperidone could be a promising candidate to apply this therapeutic approach to treat pertussis. However, the severity of this disease should be considered carefully when balancing the potential benefits of treatment with the possible adverse effects, which should be addressed in future work, for example, in in vivo studies.

We previously showed that derivatives of the approved drug cyclosporine A (CsA) inhibit the intoxication of cells with C2 toxin and PT and reduce leukocytosis in an in vivo infection model of pertussis [36,41,42]. CsA is used as an immunosuppressive drug to prevent organ rejection after transplantation through binding to Cyps and the subsequent interaction and inactivation of calcineurin [72]. CsA derivatives have been developed that inhibit Cyps without causing immunosuppression. This has opened up the possibility of using these derivatives in the context of bacterial infections. Additionally, it has been demonstrated that the simultaneous inhibition of Hsp90, Hsp70, Cyps, and FKBPs results in a more potent inhibition of clostridial binary toxins and allows for a reduction in the concentration of the inhibitors, potentially improving their adverse effects profile [39,40]. A similar strategy could be employed in future studies by combining domperidone with non-immunosuppressive derivatives of CsA to achieve a more effective therapeutic approach.

In the present study, it was demonstrated that domperidone inhibits the intoxication of cells by C2 toxin and PT in a comparable way to the established Hsp70 inhibitor VER, and this inhibition occurs through the same underlying mechanism. These findings improve our understanding of the mode of action of domperidone and could potentially serve as a starting point for the development of new therapeutic strategies based on the targeted pharmacological inhibition of Hsp70, which is crucial for the effective cellular uptake of C2 toxin and PT.

## 4. Materials and Methods

### 4.1. Protein Expression and Purification

Recombinant proteins were expressed and purified as described before: PTS1 and Gαi [73], C2I and C2IIa [3,74], Hsp70/Hsc70 [75], and Hsp90 [76].

### 4.2. Cell Culture and Intoxication Experiments

HeLa cells were obtained from DSMZ Braunschweig and cultivated in minimal essential medium (MEM) supplemented with 10% fetal calf serum (FCS), 1% penicillin–streptomycin, 1 mM Sodium Pyruvate, 0.1 mM MEM NEAA (all from Thermo Fisher Scientific, Waltham, MA, USA) and 2 mM L-glutamine (Biochrom GmbH, Cambridge, UK) at 37 °C and 5% CO_2_. Chinese hamster ovary cells strain K1 (CHO-K1) were obtained from DSMZ and maintained in DMEM and HAM’s F12 containing 5% heat-inactivated fetal calf serum, 1 mM sodium-pyruvate, and penicillin–streptomycin (1:100) at 37 °C and 5% CO_2_. Human epithelial colorectal adenocarcinoma cells (CaCo-2) were obtained from ATCC and cultured in DMEM plus 10% FCS, 1 mM sodium pyruvate, 0.1 mM non-essential amino acids, and 100 U/mL of penicillin and 100 μg/mL of streptomycin at 37 °C and 5% CO_2_. Human lung adenocarcinoma cells (A549) were purchased from ATCC and cultivated at 37 °C and 5% CO_2_ in DMEM supplemented with 10% FCS, 1 mM sodium pyruvate, 0.1 mM non-essential amino acids, and 100 U/mL of penicillin and 100 μg/mL of streptomycin. Cells were trypsinized and reseeded in a 10cm culture dish every two to three days for at most 25 cycles. Cells were detached using trypsin and reseeded every two to three days, totaling at most 15–20 times.

For the intoxication assays, cells were seeded in 24-well plates and grown for 2 days. The medium was exchanged with fresh medium containing the respective specific pharmacological inhibitors VER (inhibitor of ATP binding site of Hsp70, Hsc70, and Grp78; Merck Sigma, St. Louis, MO, USA) and domperidone (inhibitor of Hsp70; Merck Sigma) at indicated concentrations, and the cells were incubated at 37 °C and 5% CO_2_ for 30 min. Then, C2 toxin was added to indicated samples and further incubated at 37 °C and 5% CO_2_. To analyze morphological changes in cells, images were acquired on a Zeiss Axiovert 40CFL microscope with a Jenoptik ProgRes C10 CCD camera or Leica DMi1 microscope connected to a Leica MC170 HD camera (both Leica Microsystems GmbH, Wetzlar, Germany). Morphologically changed cells (i.e., cell rounding), which serve as a specific endpoint of intoxication, were counted from the pictures (four independent experiments, three pictures per well, two wells per condition) using Neuralab software v1.1 (Neuralab.de (accessed on 10 May 2023)), and the percentages of rounded cells were calculated.

### 4.3. Sequential ADP-Ribosylation of Gαi or G-Actin in Lysates from Toxin-Treated Cells

Cells were pre-incubated with respective inhibitors and then treated with PT (10 ng/mL, Merck Sigma) or C2 toxin for given incubation periods. Cells were washed with PBS 3 times and were subsequently frozen at −20 °C overnight. Lysed cells were scraped off in 30 µL ADP-ribosylation buffer (for PT: 0.1 mM Tris–HCl (pH 7.6), 20 mM DTT, and 0.1 µM ATP; for C2 toxin: 20 mM Tris–HCl (pH 7.6), 1 mM EDTA, 1 mM DTT, and 5 mM MgCl_2_) plus complete protease inhibitor (Roche), followed by incubation with 100 nM PTS1 or 30 ng C2I and biotin-labeled NAD^+^ (8.3 µM; R&D Systems, Minneapolis, MA, USA) for 40 min at room temperature (PTS1) or 37 °C (C2I) for in vitro ADP-ribosylation of Gαi, which had not yet been ADP-ribosylated during the previous intoxication. Samples were subjected to SDS-PAGE, blotted, and biotin-labeled (i.e., ADP-ribosylated). Gαi was detected with streptavidin–peroxidase (Strep-POD, Sigma-Aldrich, Merck, St. Louis, MO, USA) using the ECL system. Comparable amounts of protein were confirmed by Ponceau S staining and Hsp90 detection with a specific antibody (Santa Cruz, Dallas, TX, USA). Densitometric quantification of Western blot signals was measured using Image J, and values were normalized on the amount of loaded protein.

### 4.4. In Vitro Enzyme Activity of C2I and PTS1

HeLa cell lysate (30 µg of protein) was pre-incubated with respective inhibitors at indicated concentrations for a total of 25 µL ADP-ribosylation buffer (for PTS1: 0.1 mM Tris–HCl (pH 7.6), 20 mM DTT, and 0.1 µM ATP; for C2 toxin: 20 mM Tris–HCl (pH 7.6), 1 mM EDTA, 1 mM DTT, and 5 mM MgCl_2_) plus complete protease inhibitor for 30 min at 37 °C or left untreated as the control. Subsequently, 100 nM C2I and 10 µM biotin-NAD^+^ were added for 30 min at 37 °C. ADP-ribosylated (i.e., biotin-labeled) G-actin was detected by Western blotting. Comparable amounts of protein were confirmed by Ponceau S staining and GAPDH detection with a specific antibody (Santa Cruz). Densitometric quantification of Western blot signals was measured using Image J, and values were normalized on the amount of loaded protein.

Recombinant Gαi (0.8416 µM) was incubated for 30 min with the respective inhibitors at room temperature. As a control, Gαi was incubated with DMSO (the solvent of inhibitors). The final concentration of DMSO was compared to the highest DMSO concentration used for inhibitors. After 30 min, 100 nM PTS1 and 10 µM biotin-labeled NAD^+^ were added and incubated for 30 min at room temperature. Samples were subjected to SDS-PAGE and blotted onto a nitrocellulose membrane. Biotin-labeled (i.e., ADP-ribosylated) Gαi was detected with streptavidin–peroxidase, and the intensity of signals was quantified by densitometry using Image J. Comparable amounts of protein were confirmed by Ponceau S staining.

To test the effect of chaperones on PTS1 enzyme activity, recombinant PTS1 (100 nM) was incubated at RT or 37 °C for 15 min. Meanwhile, ATP (50 µM/1 mM; Merck Sigma) and chaperones (200 nM Hsp90/Hsp70/Hsc70) were pre-incubated for 15 min at RT or 37 °C before being added to PTS1 and further incubated. After 30 min, recombinant Gαi (0.8416 µM) was added together with 10 µM biotin-NAD^+^ and incubated for 30 min at RT or 37 °C. Biotin-labeled (i.e., ADP-ribosylated) Gαi was detected by Western blotting.

### 4.5. Binding of C2 Toxin and PT to Cells

To test the effect of inhibitors on binding of C2 toxin, cells were pre-incubated with respective inhibitors for 30 min at 37 °C. Then, cells were incubated on ice for 15 min followed by addition of C2 toxin on ice for 40 min. Cells were washed twice with cold PBS to remove unbound toxin. PBS was removed, and the plate was frozen at −20 °C to enable cell lysis. A total of 30 µL of ADP-ribosylation buffer plus protease inhibitor were added to each well. Cells were scraped and transferred to reaction tubes. Biotin-NAD^+^ was added for 10 min at 37 °C to allow ADP-ribosylation and therefore biotin-labeling of G-actin in lysates by the bound enzyme component C2I. Samples were analyzed by SDS-PAGE and Western blotting, detecting biotin-labeled G-actin. Intensity of signal was quantified by densitometry using Image J and comparable loading was confirmed by GAPDH staining.

To test the effect of inhibitors on binding of PT, cells were pre-incubated with respective inhibitors for 30 min at 37 °C followed by incubation on ice for 15 min. PT was added for 40 min on ice. After washing twice with PBS, Laemmli buffer plus DTT was added to each well. Samples were analyzed by SDS-PAGE and Western blotting, detecting PTS1 with a specific antibody (Santa Cruz, #sc-57639 (63.1G9)). Intensity of signal was quantified by densitometry using Image J, and comparable loading was confirmed by Hsp90 staining.

### 4.6. C2 Toxin Translocation Assay

The toxin translocation assay was performed as described before [3]. HeLa cells were grown for 2 days. Medium was changed and replaced with fresh serum-containing medium with or without the respective inhibitors for 30 min at 37 °C. BafA1 was added to all wells to prevent uptake of C2I into the cytosol via translocation from acidified endosomes. Cells were put on ice for 10 min, followed by addition of C2 toxin on ice for 30 min, allowing binding but not endocytosis. Medium was exchanged to acidic medium with a pH of 3.8 for 10 min to trigger pore formation into the cytoplasmic membrane and translocation of C2I directly into the cytosol. Acidic medium was removed, and cells were further incubated in serum-containing medium with BafA1 at 37 °C. Cell morphology was monitored.

### 4.7. Fluorescence Microscopy

CHO cells were seeded in ibidi 8-well plates. Medium was replaced with inhibitor containing medium or medium without inhibitor as the control, and cells were incubated for 30 min at 37 °C. PT was added for 4 h. Then, cells were washed 3 times with cold PBS, fixed with 4% PFA (20 min at RT), washed 3 times with cold PBS, permeabilized with Triton X-100 (0.4% in PBS, 5 min at RT), washed 3 times with PBS, quenched with 100 nM glycine in PBS (2 min at RT), and washed 3 times with PBS. Then, cells were blocked with 10% normal goat serum (Jackson ImmunoResearch, Philadelphia, PA, USA), probed with anti-PTS1 antibody (1:200 in blocking solution, 1 h at 37 °C; Santa Cruz Biotechnology), washed 4 times with PBST, probed with fluorescence-labeled secondary antibody anti-mouse488 (1:1000 in blocking solution; Invitrogen, Waltham, MA, USA) and with Hoechst (1:10,000 in PBST for 5 min). Cells were washed 5 times. Images were taken with Keyance BZ-X810 fluorescence microscope (Osaka, Japan).

### 4.8. iGIST Bioassay

The iGIST bioassay [27] is based on HEK293 cells that ectopically express the Gαi-coupled somatostatin receptor 2 (SSTR2) GPCR and a luminescent cAMP probe (GloSensor-22F, Promega, Madison, WI, USA). The cells were seeded in 96-well plates with white walls and a translucent bottom (View-Plate 96, Perkin Elmer, Waltham, MA, USA). To initiate the assay, cells were pre-incubated with DOM for 30 min. PT (100 ng/mL) or matched buffer SolC (50% glycerol, 50 mM Tris, 10 mM glycine, 0.5 M NaCl, pH 7.5) was added to the cells for 5 h. After removal of the medium, 45 μL of inducing medium (comprising 2% GloSensor reagent (Promega), 400 μM of the phosphodiesterase inhibitor IBMX (Sigma) in DMEM/F12 medium and CO_2_-independent medium (4v of DMEM/F12 per 5v CO_2_-independent medium), supplemented with 0.1% (*w*/*v*) bovine serum albumin) was added and equilibrated for 45 min at room temperature in the dark. Baseline luminescence was recorded for 15 min with the Orion microplate luminometer (Berthold Detection Systems, Oak Ridge, TN, USA). The cells were then spiked with forskolin (10 μM, Merck Sigma, Darmstadt, Germany) to activate adenylate cyclase and octreotide acetate (20 nM, Bachem, Bubendorf, Switzerland) to activate the Gαi-coupled SSTR2 GPCR in 25 mM HEPES buffer (pH 7.4). Luminescence, which corresponds to cAMP levels in the cells, was measured for another 60 min. The GraphPad Prism software was employed to calculate the baseline-subtracted area under the curve (AUC) for the quantification of kinetic curves.

### 4.9. Reproducibility of Experiments and Statistics

All experiments were performed independently at least 3 times. Number of replicates is given in the respective figures. Representative results are shown in the figures. Western blots were cropped for display reasons only. Data were statistically analyzed as stated in the figure legends using GraphPad Prism (**** *p* < 0.0001, *** *p* < 0.001, ** *p* < 0.01, * *p* < 0.05, ns = not significant *p* > 0.05).

## Data Availability

The datasets generated and/or analyzed during the current study are either included in the study or available from the corresponding author on reasonable request.

## Supplementary Materials

The following supporting information can be downloaded at: https://www.mdpi.com/article/10.3390/toxins15070412/s1, Figure S1: Effect of different DOM concentrations on intoxication of HeLa cells with C2 toxin. HeLa cells were pre-incubated at 37 °C with indicated concentrations of DOM for 30 min or left untreated for control (con). Then, cells were challenged with C2 toxin (50 ng/ml C2I plus 100 ng/ml C2IIa) for 5 h in the presence or absence of the inhibitor. Pictures show the toxin-induced morphological changes after 5 h.
