# Supplementary material for: Domperidone Inhibits Clostridium botulinum C2 Toxin and Bordetella pertussis Toxin
Source: Toxins (Basel). 2023 Jun 25;15(7):412. doi: 10.3390/toxins15070412 (PMC10467066; doi:10.3390/toxins15070412)
Supplement: Supplementary file 1 [file toxins-15-00412-s001.zip › toxins-2430451-supplementary.pdf]

Supplementary figure S1

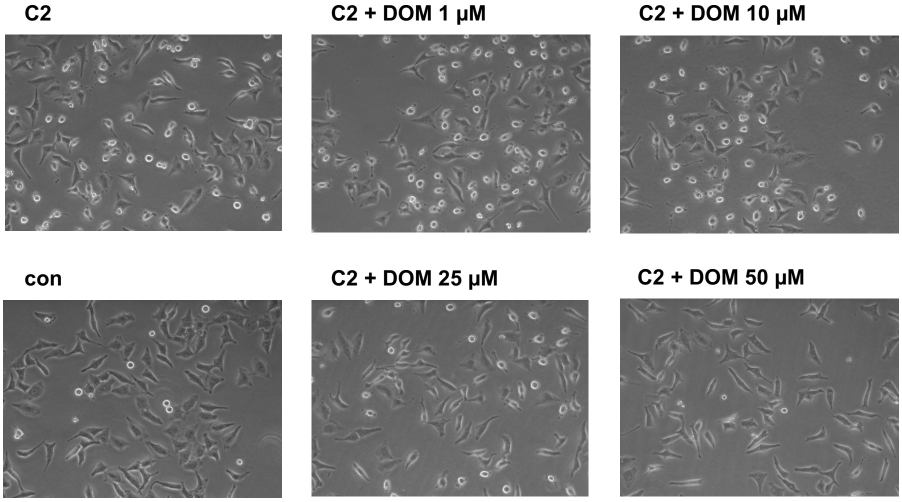

Supplementary figure S1. Effect of different DOM concentrations on intoxication of HeLa cells with C2 toxin. HeLa cells were pre-incubated at 37 ° C with indicated concentrations of DOM for 30 min or left untreated for control (con). Then, cells were challenged with C2 toxin (50 ng/ml C2I plus 100 ng/ml C2IIa) for 5 h in the presence or absence of the inhibitor. Pictures show the toxin-induced morphological changes after 5 h.
